# Sepsis after the Altemeier procedure: A case report

**DOI:** 10.1097/MD.0000000000046401

**Published:** 2026-05-12

**Authors:** Jiameng Zhu, Lin Liu, Hezhai Yin, Lidong Shen

**Affiliations:** a Zhejiang Chinese Medical University, Hangzhou, Zhejiang Province, China; b Jiaxing Hospital of Traditional Chinese Medicine, Jiaxing, Zhejiang Province, China.

**Keywords:** Altemeier procedure, anastomotic leakage, case report, rectal prolapse, sepsis

## Abstract

**Rationale:**

Anastomotic leakage represents the most critical complication following the Altemeier procedure. However, sepsis resulting from anastomotic leakage has been rarely reported in the literature.

**Patient concerns:**

A 53-year-old male with chronic obstructive pulmonary disease and rectal prolapse underwent the Altemeier procedure. On postoperative day 4, he developed sepsis secondary to anastomotic leakage.

**Diagnoses:**

The patient’s condition deteriorated after the Altemeier procedure, presenting with sepsis, anastomotic leakage, presacral infection, and acute exacerbation of chronic obstructive pulmonary disease.

**Interventions:**

Terminal ileostomy was performed, and a self-designed double-lumen irrigation-drainage catheter was placed at the anal anastomotic site for irrigation and drainage to control the source of infection. Concurrent intravenous antimicrobial therapy with meropenem and levofloxacin was administered.

**Outcomes:**

22 days after the terminal ileostomy procedure, procalcitonin and C-reactive protein levels normalized, and the patient was discharged. At the 4-month follow-up, colonoscopy revealed complete healing of the anastomosis, and successful ileostomy reversal surgery was subsequently performed.

**Lessons:**

For patients with rectal prolapse who have other comorbid infections or malnutrition, thorough preoperative assessment before the Altemeier procedure is crucial for preventing severe anastomotic leakage.

## 1. Introduction

The Altemeier procedure, also known as perineal rectosigmoidectomy, involves the resection of prolapsed rectum and a portion of the sigmoid colon through a perineal incision, followed by intestinal re-anastomosis. This approach offers advantages including minimal invasiveness and rapid recovery, making it particularly suitable for patients unfit for traditional abdominal surgery. Consequently, it stands as one of the most commonly employed surgical techniques for rectal prolapse in clinical practice.^[1]^

**Figure 1.**
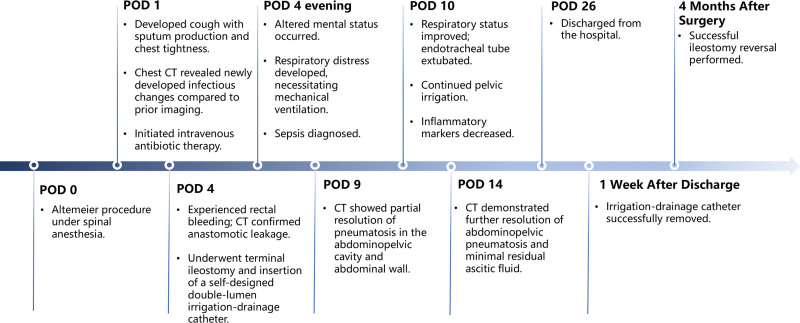
Clinical timeline.

Anastomotic leakage represents a severe complication following Altemeier procedure, with reported incidence ranging from 1.88% to 5.48%.^[2,3]^ Mild cases may be managed conservatively without surgical intervention, utilizing measures such as oral antibiotics or transanal irrigation. In contrast, severe leakage necessitates immediate surgical reintervention.^[4]^ This is one of the first detailed case reports documenting sepsis specifically linked to anastomotic leakage after the Altemeier procedure. Therefore, we present this case report detailing an instance of sepsis subsequent to the Altemeier procedure. The rapid clinical progression from anastomotic leakage to systemic sepsis is detailed in the chronological timeline (Fig. 1).

## 2. Case report

**Figure 2.**
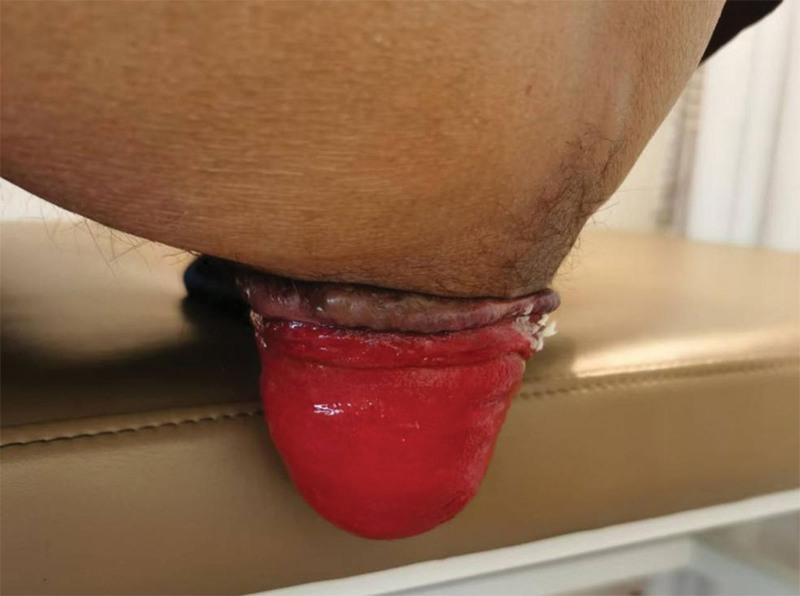
Preoperative presentation of rectal prolapse.

**Figure 3.**
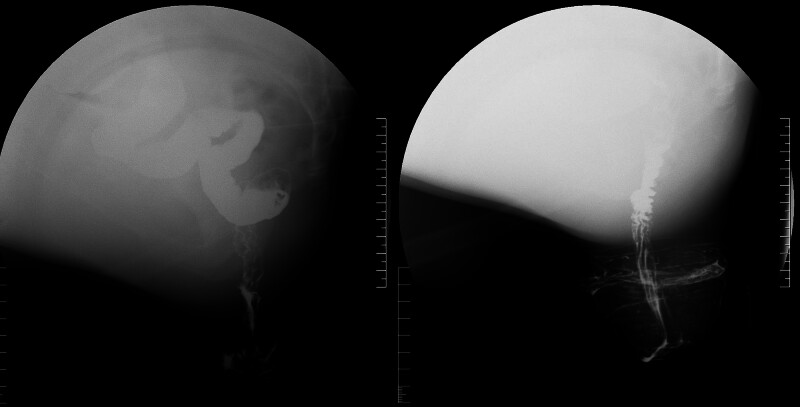
Dynamic defecography and thoracic imaging.

A 53-year-old male presented to our hospital because of sensation of anal prolapse after defecation for 3 months. 3 years ago, the patient began experiencing anal discomfort, particularly after defecation. About 3 months ago, the prolapse became circumferential after defecation and required manual reduction to reposition it. The patient also had chronic obstructive pulmonary disease (COPD) for 1 year and recently contracted pneumonia. Notably, the patient presented with significant nutritional compromise, evidenced by a body mass index (BMI) of 17.8 kg/m² and an Nutritional Risk Screening 2002 score of 4. In the squatting position, a concentric, ring-like prolapse of the rectal mucosa approximately 6 cm in length was observed (Fig. 2). Pulmonary function: Severe mixed ventilatory dysfunction with impaired diffusion capacity for carbon monoxide; pelvic magnetic resonance imaging +  diffusion weighted imaging + enhancement (3.0T): thickening of the rectal wall with rectal prolapse; chest computed tomography (CT): bronchial lesions with bilateral lung infections, local cavitary formation within the lesions, emphysema; Defecography: complete rectal prolapse with sacral-rectal separation (Fig. 3A and B).

**Figure 4.**
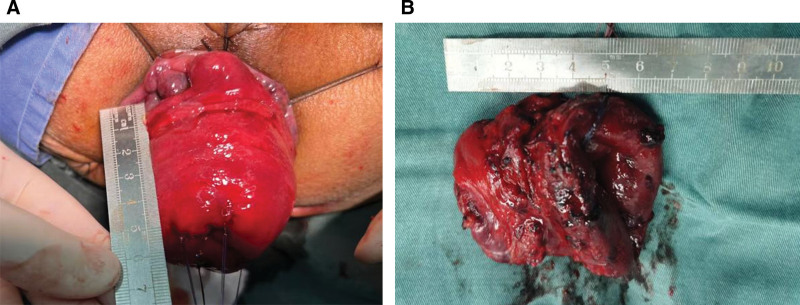
(A) Intraoperative prolapse of the anal canal. (B) Resected anal canal specimen.

**Figure 5.**
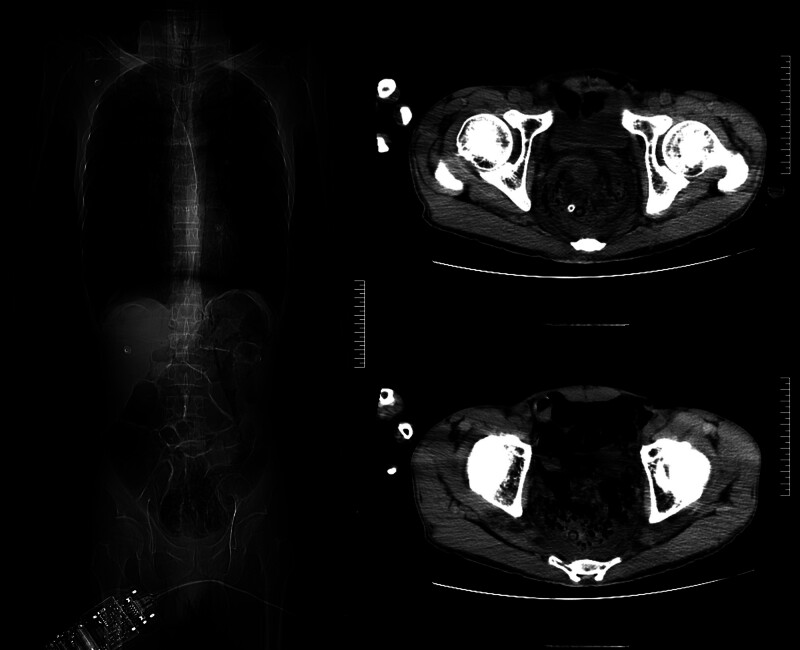
Computed tomography (CT) on January 05, 2024.

After controlling the pulmonary infection, the patient underwent an Altemeier procedure under spinal anesthesia on January 1, 2024 (Fig. 4A and B). The Altemeier procedure was performed using the standardized steps summarized in Table 1. The patient received supportive treatment including oxygen therapy, broad-spectrum antibiotics, nutritional support, and fluid resuscitation after the Altemeier procedure. On January 2, 2024, the patient developed cough with sputum production and chest tightness. Chest CT revealed bronchial lesions with bilateral pulmonary infection and cavitary lesions within local infectious foci, demonstrating newly developed infectious changes in the affected regions compared to prior imaging. Given the diagnosis of COPD with acute exacerbation and pulmonary infection, empirical antibiotic therapy was initiated with intravenous meropenem (1 g every 8 hours) combined with levofloxacin (500 mg once daily). The patient occasionally experienced dyspnea and bronchospasm, for which methylprednisolone sodium succinate was administered intravenously to alleviate airway constriction. On January 5, 2024, rectal bleeding occurred. Digital rectal examination revealed a rupture on the posterior rectal wall, and anoscopy showed localized congestion and erosion at the posterior anastomotic site, with a ruptured area approximately 2 cm in diameter. CT showed bowel distension with intraluminal gas accumulation (Fig. 5), indicating anastomotic leakage. Terminal ileostomy was performed promptly under spinal anesthesia and a self-designed double-lumen irrigation-drainage catheter was inserted at the anal anastomotic site for continuous saline irrigation. That evening, the patient developed altered mental status and respiratory distress. To improve ventilation, the patient was initiated on mechanical ventilation. Sepsis-related Organ Failure Assessment score, performed after surgery, was 8, with contributions from respiratory system (3 points), platelet count (1 point), and nervous system (4 points), while liver system, circulatory system, and renal systems showed no dysfunction (0 points each). Therefore, the postoperative diagnoses included sepsis, anastomotic leakage, presacral infection, COPD with acute exacerbation, pulmonary infection, respiratory failure, and hypoalbuminemia.

**Table 1:** Altemeier procedure’s steps.

Step number	Key procedure	Detailed description
1	Exposure of prolapse	The prolapsed bowel segment was circumferentially retracted inferiorly using non-crushing Allis forceps, fully exposing the cylindrical prolapse measuring approximately 8 cm in length.
2	Circumferential incision	A full-thickness circumferential incision was made through the outer rectal wall approximately 1.5–2 cm proximal to the dentate line using an ultrasonic scalpel. Traction sutures were placed at each quadrant of the distal rectal stump.
3	Mobilization and reduction	The rectum and sigmoid colon were inferiorly retracted. The outer prolapsed layer was everted caudally, followed by mesorectal dissection along the bowel wall with preservation of the mesorectum. The prolapsed segment was subsequently straightened under tension.
4	Pelvic dissection	The anterior pelvic floor peritoneum was incised, exposing the rectovesical pouch. Redundant pelvic peritoneal tissue was excised.
5	Irrigation and peritoneal closure	The surgical field was irrigated with dilute povidone-iodine solution. The pelvic peritoneum was closed 5–6 cm proximal to the planned anastomotic line.
6	Pelvic floor reconstruction	The rectovesical pouch was elevated superiorly. Posterior levatorplasty was performed with 2 interrupted sutures approximating the levator ani muscles.
7	Resection and anastomosis	The prolapsed bowel segment was resected. End-to-end coloanal anastomosis was completed using interrupted full-thickness sutures. The anastomotic site was reduced intra-anally. An alginate calcium-Vaseline gauze drain was inserted transanally and secured with sterile gauze dressing.

**Figure 6.**
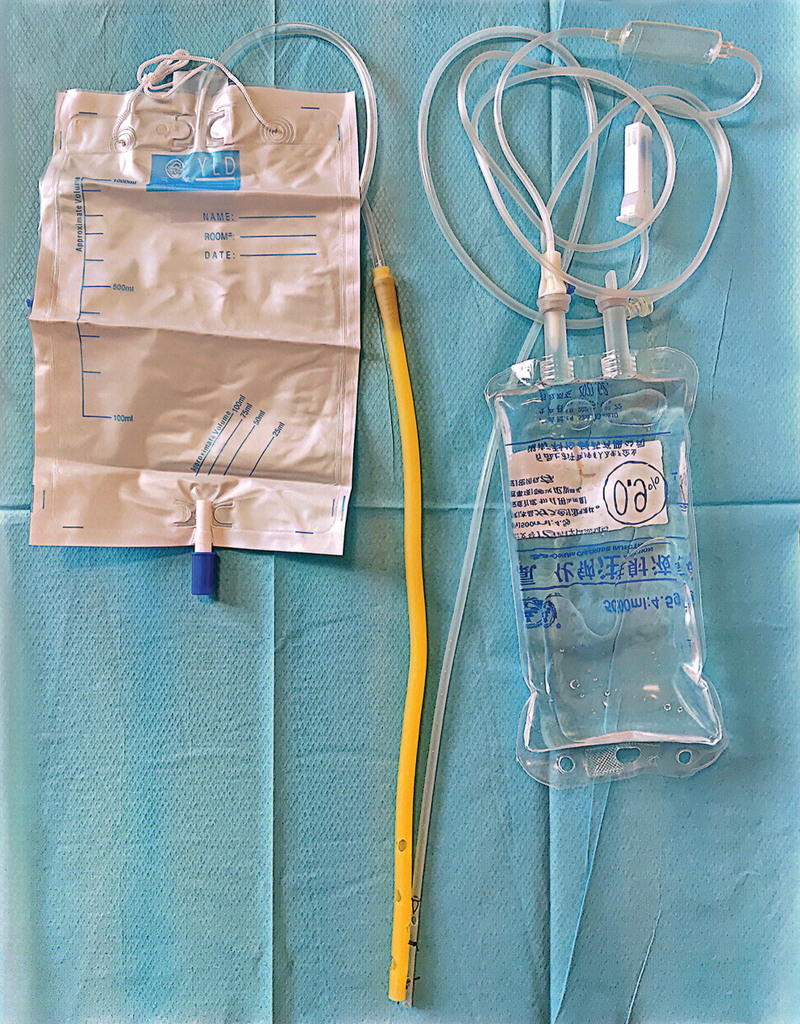
Self-designed double-lumen irrigation-drainage catheter: composed of irrigation port, drainage port and collection bag.

**Figure 7.**
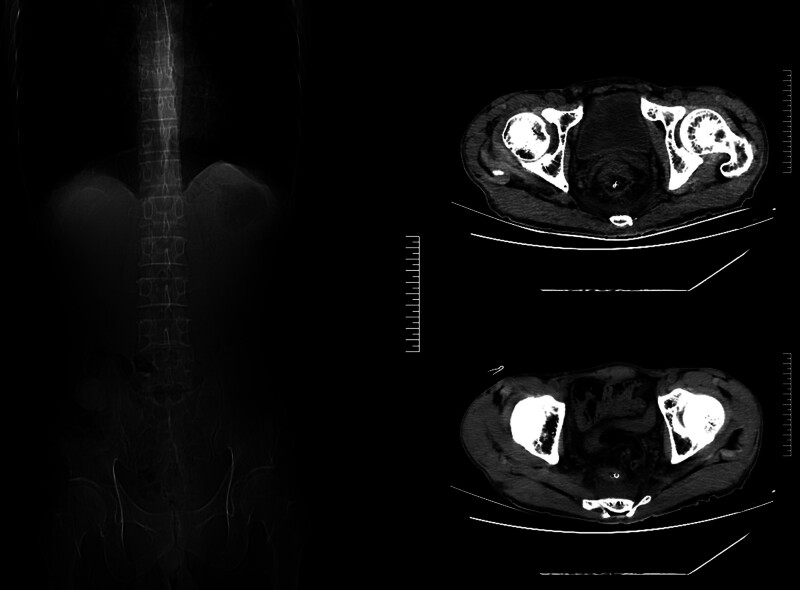
Computed tomography (CT) on January 26, 2024.

**Figure 8.**
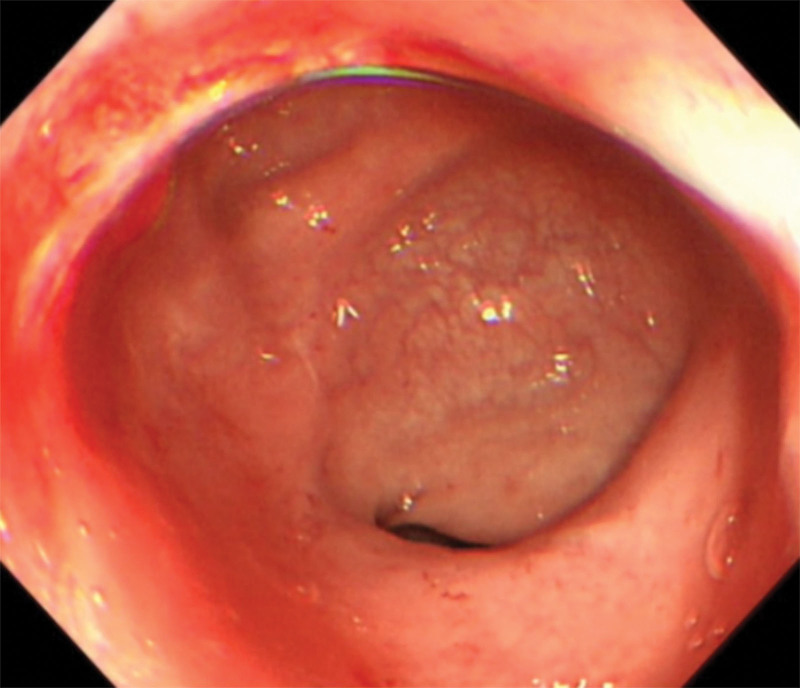
Surveillance colonoscopy at 4 months post-discharge.

Persistently elevated procalcitonin (PCT) and C-reactive protein (CRP) necessitated continued meropenem and linezolid therapy, enteral nutrition, albumin infusion, and fresh frozen plasma. The self-designed double-lumen irrigation-drainage catheter (Fig. 6) has demonstrated significant efficacy in controlling local infection. This device features separate channels for continuous saline infusion and drainage, typically maintained at an irrigation rate of 100 mL/h. When turbid drainage was observed, supplementary rapid manual irrigation via syringe was implemented. Clinical outcomes confirmed its effectiveness in reducing retained fecal debris and mitigating the local inflammatory response. As documented in Table 2, The patient became afebrile with normalized PCT and CRP levels within days, while CT showed resolved abdominopelvic and abdominal wall pneumatosis (Fig. 7). Discharge occurred on January 27, 2024, and the catheter was successfully removed during an outpatient visit 1 week later. At a follow-up visit 4 months later, colonoscopy revealed well-healed anastomosis with no stricture (Fig. 8) and ileostomy reversal was successfully performed.

**Table 2:** Procalcitonin and C-reactive protein results in patient following terminal ileostomy.

Date	PCT (ng/mL)	CRP (mg/L)
POD 1	1.35	277.96
POD 3	0.74	236.82
POD 5	1.18	62.85
POD 7	0.59	47.52
POD 9	0.39	13.40
POD 11	0.49	10.86

CRP = C-reactive protein, PCT = procalcitonin, POD = postoperative day.

Informed consent statement: Written informed consent was obtained from the patient for publication of this case report and any accompanying images.

Ethical approval was obtained from the Ethics Committee of Jiaxing Hospital of Traditional Chinese Medicine (Approval No. JXTCM-IRB-2025-088).

## 3. Discussion

As perineal procedure for the treatment of rectal prolapse, the Altemeier procedure is more suitable for patients with long-segment prolapse. Compared with the Delorme procedure (mucosal resection and rectal wall plication), the Altemeier procedure requires a full-thickness resection of the rectum followed by a low colorectal anastomosis. Therefore, the Altemeier procedure carries a higher risk of anastomotic leakage.^[3]^

The International Study Group of Rectal Cancer classifies anastomotic leakage after anterior resection of the rectum into 3 grades: Grade A (clinically asymptomatic, usually detected radiologically and requiring no intervention), Grade B (clinically symptomatic, with symptoms such as abdominal pain and fever, requiring conservative treatment such as antibiotics and drainage but not surgical intervention), and Grade C (severe, with significant clinical deterioration and usually requiring relaparotomy).^[4]^ This classification provides a valuable reference for the evaluation and management of anastomotic leakage after the Altemeier procedure. It is noteworthy that International Study Group of Rectal Cancer’s definition specifically addresses anastomotic leakage after the abdominal approach for rectal resection, while the Altemeier procedure is inherently a perineal approach. Current research mainly focuses on anastomotic leakage after abdominal anterior resection for rectal cancer. Multiple studies have shown that the risk factors for anastomotic leakage after anterior resection of the rectum for rectal cancer, including gender, age, low anastomotic level, compromised blood supply, anastomotic tension, local infection, and poor systemic nutritional status.^[5]^ However, the anastomosis for perineal approach surgery is typically situated lower, and the risk factors may also differ from those in abdominal surgery. This area currently lacks sufficient research and requires further investigation.

In this case, the patient had underlying COPD and pulmonary infection preoperatively, along with a BMI of 17.8 kg/m² and an NRS 2002 score of 4. Although the infection was controlled preoperatively, the primary infection worsened after the Altemeier procedure. By postoperative day 4, the patient developed anastomotic leakage and extensive presacral infection, ultimately leading to severe sepsis. For patients who are considered by the surgeon to be at high risk of anastomotic leakage after rectal cancer surgery, a prophylactic ileostomy is usually performed intraoperatively.^[6]^ However, for benign diseases such as rectal prolapse, a prophylactic ileostomy remains controversial and typically reserved for intraoperative technical concerns. For this patient with severe malnutrition and pulmonary infection like this, priority should have been given to optimizing nutritional status and controlling infection before surgery, or considering concurrent prophylactic ileostomy given the significant risk profile.

The differentiation of sepsis etiology was crucial in this patient. The respiratory symptoms and radiographic progression of pulmonary infection on postoperative day 1 suggested a potential pulmonary source for sepsis. However, the diagnosis of anastomotic leakage with extensive presacral infection on postoperative day 4 established a more direct and severe intra-abdominal infectious focus. The patient’s rapid clinical improvement following ileostomy and irrigation-drainage for the anastomotic leakage strongly supports that the leakage was the primary etiology of sepsis in this case. Nevertheless, a potential synergistic effect between the pulmonary and abdominal infections, exacerbating the clinical deterioration, cannot be entirely ruled out.

This report has several limitations. Although the self-designed double-lumen irrigation-drainage catheter demonstrated favorable outcomes in this case, its application requires further validation through larger studies to confirm its efficacy and safety. Additionally, the clinical manifestations of anastomotic leakage are variable. As this device was designed for the specific circumstances of a particular patient, its generalizability across different medical centers needs further evaluation.

## 4. Conclusion

This case highlights the risk of postoperative sepsis associated with the Altemeier procedure. Comprehensive preoperative assessment is imperative for high-risk patients, given that anastomotic leakage can pose life-threatening consequences. Postoperatively, close monitoring of biomarkers such as PCT and CRP is essential for early detection and intervention of anastomotic leakage. There remains a need for further clinical studies to guide treatment decisions regarding anastomotic leakage after the Altemeier procedure for rectal prolapse.

## Author contributions

**Conceptualization**: Jiameng Zhu.

**Investigation**: Lidong Shen.

**Resources**: Hezhai Yin.

**Writing – original draft**: Jiameng Zhu

**Writing – review & editing**: Lin Liu, Hezhai Yin.
